# Experimentally attacking quantum money schemes based on quantum retrieval games

**DOI:** 10.1038/s41598-019-51953-9

**Published:** 2019-11-08

**Authors:** Kateřina Jiráková, Karol Bartkiewicz, Antonín Černoch, Karel Lemr

**Affiliations:** 1 RCPTM, Joint Laboratory of Optics of Palacký University and Institute of Physics of Czech Academy of Sciences, 17. listopadu 12, 771 46 Olomouc, Czech Republic; 2 Faculty of Physics, Adam Mickiewicz University, PL-61-614 Poznan, Poland; 3 Institute of Physics of the Czech Academy of Sciences, Joint Laboratory of Optics of PU and IP AS CR, 17. listopadu 50A, 772 07 Olomouc, Czech Republic

**Keywords:** Quantum optics, Fibre optics and optical communications

## Abstract

The concept of quantum money (QM) was proposed by Wiesner in the 1970s. Its main advantage is that every attempt to copy QM unavoidably leads to imperfect counterfeits. In the Wiesner’s protocol, quantum banknotes need to be delivered to the issuing bank for verification. Thus, QM requires quantum communication which range is limited by noise and losses. Recently, Bozzio *et al*. (2018) have demonstrated experimentally how to replace challenging quantum verification with a classical channel and a quantum retrieval game (QRG). This brings QM significantly closer to practical realisation, but still thorough analysis of the revised scheme QM is required before it can be considered secure. We address this problem by presenting a proof-of-concept attack on QRG-based QM schemes, where we show that even imperfect quantum cloning can, under some circumstances, provide enough information to break a QRG-based QM scheme.

## Introduction

All payment methods are potential targets of thieves and counterfeiters. Over the course of history, we have witnessed a race of arms between the counterfeiters and issuers of various currencies. Remarkably, Sir Isaac Newton, who became the master of Royal Mint, enforced laws against counterfeiting. Nevertheless, the methods used by Newton become obsolete when it comes to modern payment methods. With the rapid technological progress, we are beginning to consider a situation where counterfeiting is no longer limited by the available technology, but rather by the laws of nature. An example of such fundamental limitation is the no-cloning theorem^1,2^, which guaranties security of quantum money^3–7^.

In a recent paper, Bozzio *et al*.^8^ reported on an implementation of a QM scheme based on QRGs^9–11^. While this result brings QM closer to practical implementation, here we demonstrate that QRG-based QM schemes are still vulnerable to a new kind of attack (for some typical attacks see ref. ^12–16^) which can be considered a quantum version of sniffing (a hacking method used to monitor clasical information). The general idea of our attack can be used against a broader range of QM schemes based on QRG^17–19^ and potentially on other quantum communication protocols. Thus, our results can facilitate future practical implementations of QM by providing a method for exploring the security limits allowed in QRG-based protocols. For the purpose of our research we have experimentally recreated the original scheme of ref.^8^. Its working principle can be described as follows: the bank encodes QM (as a quantum token) using a secret sequence of qubit pairs chosen from the list of eight options:

1 $$S=\{|0+\rangle ,|0-\rangle ,|1+\rangle ,|1-\rangle ,|+0\rangle ,|-0\rangle ,|+1\rangle ,|-1\rangle \}\,,$$

where |0〉, |1〉 are logical qubit states, and $|\pm \rangle =\frac{1}{\sqrt{2}}(|0\rangle \pm |1\rangle )$ stand for their superpositions. The tokens and their serial number are then stored on a quantum credit card^8,20,21^ subsequently given to a client of the bank. Upon payment, the credit card is inserted into the vendor’s terminal which is supposed to perform projection measurements on these pairs in a measurement basis requested by the bank (randomly chosen to be either 0/1 or +/− for an entire pair). Then, the terminal sends the classical outcomes of those measurements to the bank. The main advantage of this scheme is that the terminal measurement itself is sufficient for authentication of the credit card, so quantum states do not have to be sent to the bank for verification. The bank just checks the results knowing the specific encoded states and either accepts or denies the payment. A small amount of errors is expected to appear in the verification procedure to account for implementation imperfections. The acceptable amount of errors needs to be small enough to ensure that payment by a cloned quantum credit card is denied. In contrast to the original Wiesner QM scheme^3^, no on-line quantum channel has to be used for payment. Thus, the verifiability problem as defined by Aaronson and Christiano^22^ is at least partially solved.

This protocol is secure against a dishonest terminal only if each quantum sequence is generated using a truly random encoding. However, such condition would give rise to a giant database problem, as discussed in^22^ and^23^. The random sequence approach is highly impractical or even infeasible. In practice, there has to be one secret encoding function shared by a certain number of quantum banknotes or tokens (i.e., sequences of quantum states and their serial numbers). Hence, in our research we test limitations of sharing a secret encoding by multiple tokens. The tokens are therefore encoded using a prescription based on the output of a classical algorithm. Inputs to this algorithm are the publicly known serial numbers (SN) and secret salt (a secret number).

The aim of suggested attack is not to copy single banknotes but to be able to generate new banknotes that pass as genuine. Note that by employing the studied attack strategy, a terminal can collect in principle unlimited data during its operation. This attack can be run in parallel while having many wiretapped terminals. Moreover, we show that by using optimal quantum cloning we can learn the secret faster than by limiting the attack only to classical data processing.

**Figure 1:**
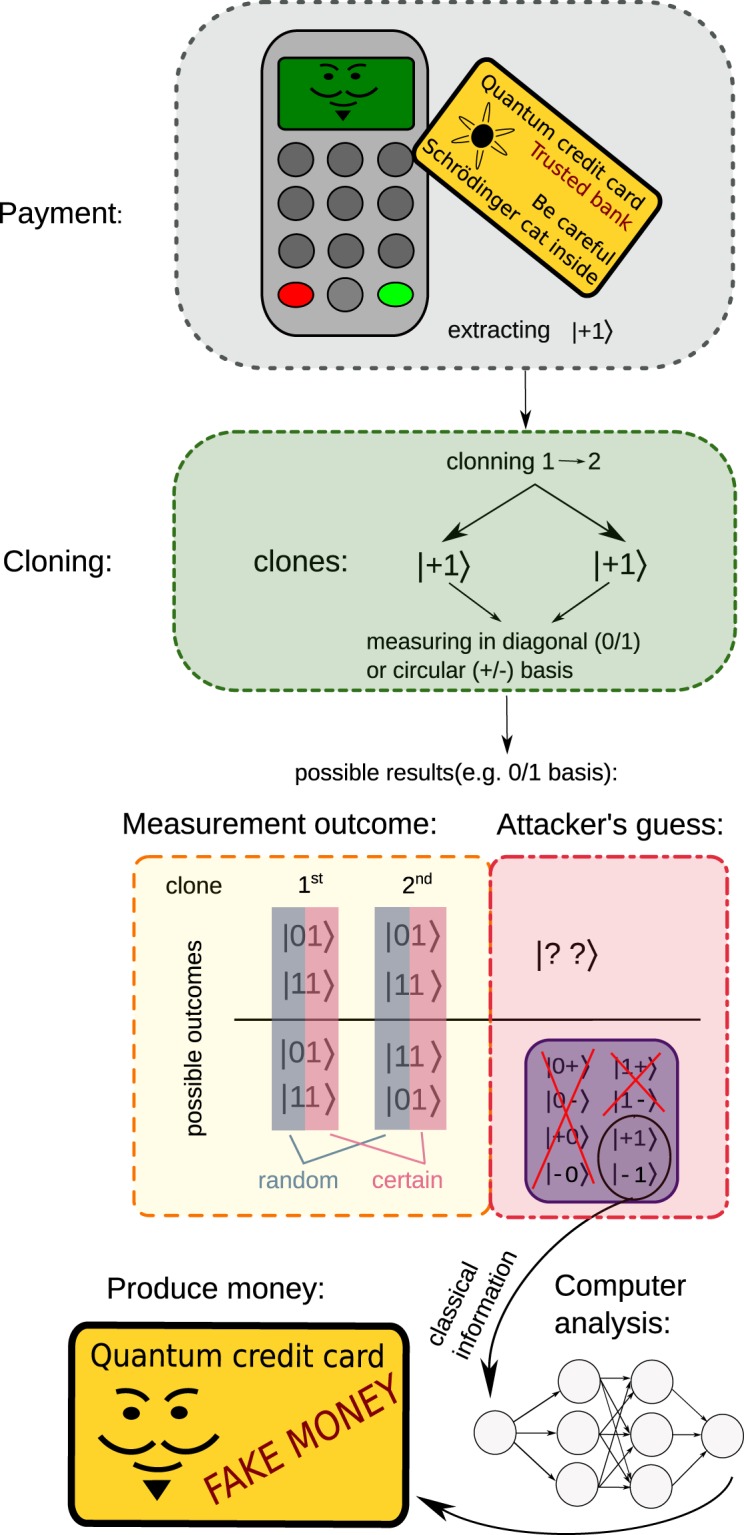
Attack on a quantum credit card utilising a hacked terminal. During a transaction a pair of states (e.g., |+1〉) is extracted from the card and cloned. Here, for simplicity, we depict only the situation where all the qubits are perfectly copied (the probability of such event is proportional to *F*^2^). Then, measurements are performed on all four copies in the basis randomly chosen by the bank (e.g. 0/1). If the measurements on copied qubit pairs produce one of two results from the bottom block of the table of outcomes, the attacker learns the originally encoded state (in this case |?1〉). This procedure is repeated until a relation between the quantum states and serial numbers is learned. Since then, the attacker can issue perfectly counterfeit quantum credit cards.

Although quantum cloning has been already used to counterfeit QM^4^, the purpose of quantum cloning here is completely different and as such is virtually undetectable by the bank because we copy only parts of quantum tokens (i.e., quantum sequences). In terms of QRG-based QM protocol, the attacker utilises a compromised payment terminal enabling quantum cloning of an input qubit (see Fig. 1). The terminal performs measurements on both copies of a qubit providing the attacker with some information on the encoding used by the bank, if two consecutive qubits from a sequence are cloned. The frequency of cloning can be arbitrarily small and therefore made unrecognisable from noise. After gathering enough data, the attacker reveals the secret encoding used by the bank for preparing credit cards. Since then, they can issue fake quantum credit cards indistinguishable from the original ones issued by the bank.

Quantum cloning has been proposed and tested as a means of attack on quantum communications protocols^12–14,24,25^. There is, however, a significant conceptual difference between cloning attack on quantum cryptography and the quantum money scheme discussed in this paper. The necessary condition for successful attack on quantum cryptography protocol is having ideally 100% of the quantum key eavesdropped. Otherwise, the security can be attained by privacy amplification arbitrarily lowering the attacker’s probability of decoding the shared message^26^. On the other hand, attack on QM based on QRG described in this paper only requires to clone a small fraction of the money tokens. Such infrequent cloning is basically undetectable in the noise, albeit gathering data would proceed slowly. A typical obstacle in cloning-based QM attacks is requirement of high cloning success rate as at least half of the token needs to be cloned successfully (i.e. not destroyed)^4^. This fact needs to be dealt with on probabilistic platforms such as linear optics. The method discussed in this paper is completely free of this limitation.

## Results

We have implemented the quantum sniffing attack on the platform of linear optics, where qubits are encoded as polarisation states of single photons. The optimal cloning strategy (i.e., maximizing single-copy cloning fidelity) for copying qubits from the set *S* is implemented as the symmetric phase-covariant cloning (SPCC)^4,12,27^. In the experiment, pairs of input qubits |*ψ*_1_*ψ*_2_〉_in_ ∈ *S* were subjected to SPCC procedure obtaining two clones ${\hat{\varrho }}_{{\rm{1A}}}\otimes {\hat{\varrho }}_{{\rm{2A}}}\,{\rm{and}}\,{\hat{\varrho }}_{{\rm{1B}}}\otimes {\hat{\varrho }}_{{\rm{2B}}}$ of the input qubit pair. These clones were then measured in the same but random basis. In a QRG-based QM protocol the basis is selected by the bank. Due to limitations of linear optics based implementations of quantum cloners^28^, the SPCC process is probabilistic and sometimes it fails to deliver the clones. The probability of successful cloning of one input qubit is denoted *P*. Therefore the probability of cloning the entire qubit pair is *P*^2^. Quality of the clones is expressed in terms of fidelity *F* defined as $F={F}_{ij}{=}_{{\rm{in}}}{\langle {\psi }_{i}|{\hat{\varrho }}_{ij}|{\psi }_{i}\rangle }_{{\rm{in}}}$, where *i* = 1, 2 and *j* = A, B denote the first and the second clone, respectively. The probability of finding both clones ${\hat{\varrho }}_{i{\rm{A}}}$ and ${\hat{\varrho }}_{i{\rm{B}}}$ in a given state |*ψ*_*i*_〉_in_ reads *F*^2^. An example of an attack on a particular qubit pair is shown in Fig. 1.

The theoretical limit for SPCC fidelity^27^ is $F=\frac{1}{2}(1+\frac{1}{\sqrt{2}})\approx 0.854$ and on the platform of linear optics the cloning succeeds with probability $P=\frac{1}{3}$. While the limit on fidelity is fundamental in its nature, *P* depends on the physical platform used in a given implementation and can be arbitrarily close to 1. However, even on the platform of linear optics, it is possible to clone at arbitrarily high values of *P* but at the expense of reaching lower than optimal fidelity *F* (see hybrid quantum cloners^12,29^).

The terminal registers two measurement outcomes per input qubit corresponding to the clones. If the two clones of one input qubit yield identical results, while for the other yield opposite results, the attacker gains information about the encoding. With the probability *P*_*tot*_ = *P*_c_ + *P*_e_ the attacker eliminates six of the original eight encodings (see Eq. 1). One of the two remaining encodings have actually been used by the bank. The probability of obtaining correct information from the attack is ${P}_{{\rm{c}}}=\frac{1}{2}{P}^{2}{F}^{2}$, whereas ${P}_{{\rm{e}}}=\frac{1}{2}{P}^{2}{\mathrm{(1}-F)}^{2}+{P}^{2}F\mathrm{(1}-F)$ stands for the probability of getting an erroneous result due to limited cloning fidelity. Similarly, if the two clones of each input qubit yield identical results, the attacker knows that only one of four encodings might have been sent by the bank.

The attacker is able to learn the method of encoding tokens by accumulating measurement results provided that the fidelity is $F\ne \frac{1}{2}$. The cloning operation inherently introduces errors in the measurement outcomes^1,2^. Hence, the terminal might send to the bank incorrect results. If the error rate surpasses a given limit (25% in ref.^8^), the bank will reject the payment. Thus, it is necessary to introduce a strategy of attack considering all circumstances of the measurement (i.e., if cloning failed or not) and its outcomes to minimise the error rate. There are generally three distinct strategies: (i) to provide the bank with measurement outcome every time cloning takes place and even if it fails, send a random value, (ii) to send measurement outcome, only if it is registered by the terminal and report a lost qubit when cloning fails and (iii) to measure qubits after their extraction from the credit card in given measurement basis but do not perform cloning at all.

To quantify the correlations between the attacker and the genuine token we use mutual information *I*_sec_, which expresses how many bits of information can the attacker obtain upon cloning one qubit pair. The exact value of mutual information depends on the strategy used, cloning success probability *P* and fidelity *F*. In case of the third strategy (without cloning), its value is $\tfrac{1}{2}$. For more details on this strategy refer to section Methods.

Simultaneously, we denote $\epsilon$ the probability of an error being reported to the bank. The expressions for error rates $\epsilon$ for the two above-mentioned strategies can be obtained by direct calculations based on analysis of probabilities of all possible scenarios and read

2 $${\epsilon }_{(i)}=\tfrac{1}{2}\mathrm{(1}-P)+P\mathrm{(1}-F),$$

3 $${\epsilon }_{(ii)}=1-F\mathrm{.}$$

Equation (2) takes into account two situations. In the first case, one or both qubits are lost during cloning and, therefore, random results are reported to the bank (50% chance of error). In the second case, even if cloning succeeds, non-unit fidelity may cause the measurement to yield an incorrect result. The error rate in case of strategy (ii) depends only on imperfect cloning fidelity.

**Figure 2:**
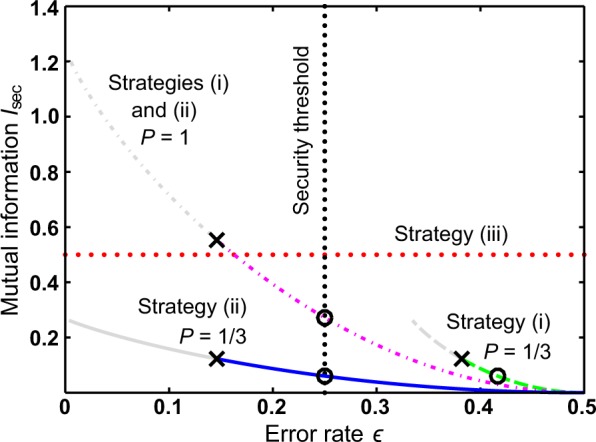
Mutual information *I*_sec_ versus error rate $\epsilon$ for two fixed probabilities $P=\{\frac{1}{3}\mathrm{;1}\}$. Vertical black dotted line represents error rate associated with security threshold discussed in refs^17,18^. Crosses mark the smallest average error introduced by optimal cloning for a fixed value of *P*. Error rates below these optimal values cannot be reached by any physical operation (greyed curves). Circles stand for limit of classical copying (*F* = 0.75). Thus, the segments of curves between circles and crosses mark the regime of quantum copying. It follows from Eq. 3 that classical copying limit in strategy (ii) always corresponds to intersection between the relevant curve and the security threshold. For more details on strategy (iii) refer to section Methods.

**Figure 3:**
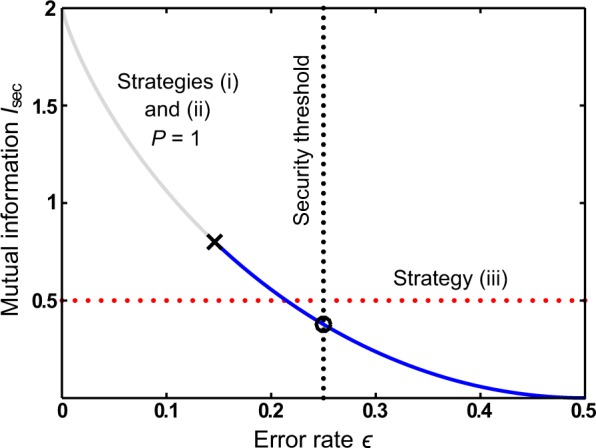
Conditional mutual information *I*_sec_ versus error rate $\epsilon$. Strategies (i) and (ii) are equal in this case.

The relation between mutual information *I*_sec_ (between the bank and the attacker) and the error rate $\epsilon$ for all strategies is shown in Fig. 2. In the figure, quantities *I*_sec_ and $\epsilon$ are functions of cloning fidelity for $\frac{1}{2}\le F\le 1$ for two cloning success rates $P=\frac{1}{3}$ (linear optics limit^4,28,29^) and *P* = 1 (deterministic cloning^4,29–31^). In case of deterministic cloning the two attack strategies coincide, but for probabilistic cloning the second strategy provides better results. It is fair to note that the mutual information of any simple linear-optical cloning strategy is lower in comparison with the no-cloning strategy (iii). On the other hand, with deterministic cloning, one can reach even higher values of mutual information and therefore cloning strategies need to be considered for security implications. Additionally, machine learning-based algorithms may require data with as little noise as possible even at the expense of the overall quantity. Post-selection on successful cloning events allows to distil such sample. Corresponding conditional mutual information yields a significantly higher value when both qubits are successfully cloned than for the no-cloning strategy (iii) (Fig. 3).

To prove the working principle of the quantum sniffing attack, let us consider a specific encoding of the quantum tokens and demonstrate the attacker’s approach to learning the encoding. Here, we assume that the bank uses a hash function to encode the tokens. Since the hash functions have become a worldwide standard for encryption and basis of many classical cryptosystems they would be easily deployable by the bank. Hash functions are designed to return very distinct results even for similar inputs making their output unique. Another advantages are, for instance: irreversibility, (i.e. impossibility to retrieve original message from a given hash), or their repeatability (they yield the same hash for the same message).

The input can be additionally modified by using a specific secret number (salt). In this case the hash function is often referred to as salted. For simplicity, let us now assume that the hash function is known to the attacker, but the salt is secret. For each token passing through the terminal, the attacker calculates hashes (outputs of the hash function) of its serial number salted by numbers from a certain range. This way the attacker investigates various encodings each corresponding to one secret number (or salt). Using the information gained by quantum sniffing, the attacker calculates the number of agreements (matching qubit pairs) between the predictions of the tested encoding and the measurement outcomes on real tokens. The encoding with highest number of agreements is most probably the one used by the bank, hence the one corresponding to the correct salt.

**Figure 4:**
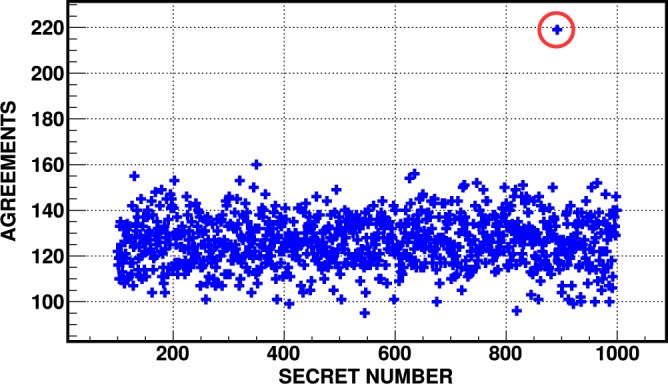
Dependence of number of agreements on all possible three-digit secret numbers evaluated for 4 040 successfully cloned photon pairs. The revealed secret number (salt) is marked by a red circle.

To showcase the attack, we have implemented token encoding using several known hash-based functions, i.e. HMAC-MD5^32^, HMAC-SHA512, HMAC-SHA256, and HMAC-SHA1 (HMAC–Hash-based Message Authentification Code^33^). Typical example of encoding using SHA512 is depicted in Fig. 4. In our proof-of-concept experiment, the salt has been sought only among three-digit numbers. To distinguish the secret number from noise originating from random matches, a sample of 4 040 successfully cloned photon pairs (corresponding to 101 serial numbers used in the experiment) has been evaluated. To optimise the computational resources of the attacker, the algorithm gradually refines the set of evaluated secret numbers. Periodically it removes secret numbers with low number of agreements from the list of evaluated numbers. Once the number of agreements for one secret number surpasses the average number of agreements by selected multiple of standard deviation, the algorithm ends and returns that number. Note that due to some error tolerance, the attacker does not necessarily need to recreate the original hash function. It would be enough if they found a function which error rate is below the security threshold.

The size of HMAC output of all used hash functions was set to be 40 bytes. As a consequence, the number of tokens necessary for guessing the secret number was independent on the number of digits of their serial number. For each hash function we have established how many photon pairs need to be successfully cloned in order to reveal the secret number with sufficient certainty. The results are summarised in Table 1. The number of cloned pairs needed does not scale with the length of the salt. The salt length only increases the classical computing time. According to our numerical simulation, number of photon pairs necessary for correct guess is linearly increasing with the number of output hash bits. However, with the length of output hash the frequency of cloning (number of cloned pairs/total number of transmitted photon pairs) does not change because the length of the token is also increasing. The output hash and the token have to have the same length in order to avoid incidents such as two inputs to the hash function yielding the same output. Longer hash output would, therefore, result in increase of computer search time, however, it would not prevent the attacker from retrieving the secret number since the searching process is performed in parallel with the cloning attack. Note that these results were obtained using our experimental results where the average cloning fidelity was found to be above 80%.

**Table 1:** Minimal number of photon pairs cloned for correct guess of the secret number (salt).

Hash-based function	Number of pairs
HMAC-MD5	1 400 ± 16
HMAC-SHA512	1 192 ± 14
HMAC-SHA256	1 060 ± 14
HMAC-SHA1	1 272 ± 13

**Figure 5:**
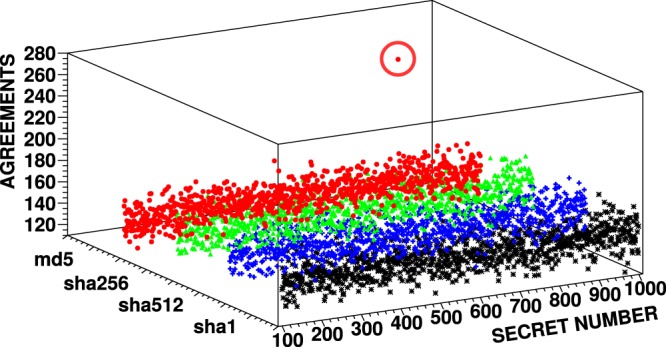
Dependence of number of agreements on all three-digit secret numbers. Four different hash functions are tested. The bank used MD5 for encoding. In this plot, 4 040 successfully cloned photon pairs were analysed. The revealed secret number (salt) is marked by a red circle.

We have also performed a generalised attack in which the attacker did not know what hash function had been used for encoding. The attacker only assumes the hash function is one from a given set. In this situation, the attacker has to calculate hashes using all hash functions in this set to encode serial numbers and count numbers of agreements as described above. The plot in Fig. 5 shows the search for the secret number among four hash functions. The tokens were encoded using MD5. Our results indicate that the correct secret number and hash function can be revealed assuming the hash function is a member of a finite set. The size of which is limited by the available time and computing power.

## Methods

Photonic qubits were encoded as four polarisation states located on the equator of Poincaré sphere: |*D*〉, |*A*〉, |*R*〉 and |*L*〉 (i.e. diagonal linear, anti-diagonal linear, right-handed and left-handed circular polarisations). Thus, the set of possible qubit pairs (1) is given as

4 $$S^{\prime} =\{|DR\rangle ,|DL\rangle ,|AR\rangle ,|AL\rangle ,|RD\rangle ,|LD\rangle ,|RA\rangle ,|LA\rangle \}\,.$$

**Figure 6:**
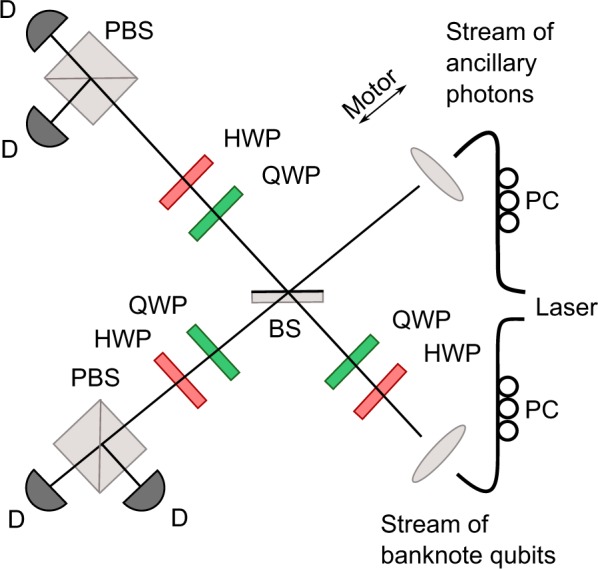
Laboratory setup for the quantum sniffing experiment. The setup operates as the compromised terminal from Fig. 1. Its components are labelled as follows: BS–partially polarising beam splitter, QWP–quarter-wave plate, HWP–half-wave plate, PBS–polarisation beam splitter, PC–polarisation controller, D–single-photon detector.

Experimental setup used in our experiment is shown in Fig. 6. Photon pairs at *λ* = 710 nm are generated in a process of type-I spontaneous parametric down-conversion (SPDC) in a BBO (*β*-BaB_2_O_4_) crystal. The crystal was pumped by Paladine (Coherent) laser operating at *λ* = 355 nm. One photon from each SPDC-generated pair served as one qubit of the cloned banknote. We used a sequence of half and quarter wave plates (HWP and QWP, respectively) to implement encoding. The second photon from the SPDC-generated pair was meanwhile used as a cloning ancilla (kept horizontally polarised as it is the theoretically known optimum for SPCC).

Given the nature of the attacked scheme, phase-covariant cloning is the optimal form of cloning attack. It has been used to attack distinguished quantum cryptography protocols such as BB84^34^ or RO4^35,36^. The attacked QM scheme uses equatorial qubits in the state

5 $$|{\psi }_{s}\rangle =1/\sqrt{2}(|0\rangle +{e}^{i\eta }|1\rangle )\,,$$

where |0〉 and |1〉 denote logical qubit states and *η* the phase. For this class of states, the phase-covariant cloner reaches fidelity of 0.854. Equatorial states can be unitarily transformed into states laying on the intersection of Bloch sphere and the plain running through the centre of the sphere for which the optimal cloning transformation is defined in Eq. 6.

Cloning is performed by an unbalanced polarisation-dependent beam splitter (BS) which implements the optimal SPCC process (for detailed theoretical description see refs^27,28,37^, for experimental implementation see also ref.^38^). Particular splitting ratio for horizontal and vertical polarisations accounted for 0.21 and 0.79, respectively. During the experiment signal and ancillary photons overlap at the BS which results with success probability of $\tfrac{1}{3}$ in the cloning transformation:

6 $$\begin{array}{rcl}\mathrm{|0}{\rangle }_{{\rm{in}}}|{\psi }_{a}\rangle  & \to  & \mathrm{|00}\rangle ,\\ \mathrm{|1}{\rangle }_{{\rm{in}}}|{\psi }_{a}\rangle  & \to  & \frac{1}{\sqrt{2}}\mathrm{(|01}\rangle +\mathrm{|10}\rangle ),\end{array}$$

where |*ψ*_*a*_〉 denotes the state of ancilla.

Subsequently, each photon is projected in the D/A or R/L measurement basis as requested by the bank (using HWPs, QWPs, and polarisers). The process of cloning is successful only if each photon leaves BS by different output port. Therefore, we are interested in coincidences between both output arms. The detection is handled by single-photon detectors operating with detection efficiency of around 60% and subsequent electronics. In the experiment, we have registered individual coincident detections one by one thus genuinely implementing the protocol described in the text.

**Figure 7:**
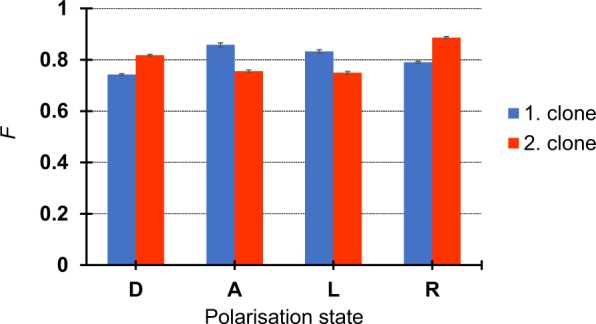
Average fidelity of the first and second clone of a qubit from the cloned set measured by projections in appropriate bases.

Quality of the clones was quantified by fidelity for both clones and each possible sequence qubit state (Fig. 7) by evaluating statistics of observed individual coincidence events. The average cloning fidelity was calculated to be (80.3 ± 0.3)% while some clones in the two output arms had slightly different fidelities. Typical detection rate was 120 pairs per second.

In order to quantify the correlation between the attacker and the information encoded as a pair of qubits, we enter the value of mutual information *I.* This value determines how many bits of information an attacker can get after cloning one pair of qubits and depends on the strategy used, success probability of cloning *P* and its fidelity *F*. Mutual information is calculated as

$$I=\mathop{\sum }\limits_{X,Y=000}^{111}\,{p}_{X,Y}{\log }_{2}\frac{{p}_{X,Y}}{{p}_{X}{p}_{Y}},$$

where ${p}_{X}={\sum }_{Y\mathrm{=000}}^{111}\,{p}_{X,Y},$ ${p}_{Y}={\sum }_{X\mathrm{=000}}^{111}\,{p}_{X,Y},$ and *X*, *Y* = 000, 001, 010, 100, 110, 101, 011, 111. The technical details on calculating probability distributions needed for calculating mutual information for all the considered strategies are given in the Supplement. Here we provide a brief introduction into the working principle of strategy (iii). Without performing quantum cloning, the attacker measures the qubits as requested by the bank and simultaneously uses this information to obtain some knowledge about the encoding used. While this approach enables to rule out some of 8 encodings, these eliminated encodings depend on the order of encoding bases. The attacker can assume that the order of encoding bases for the received qubit pair is either *Z*/*X* or *X*/*Z*, where *Z* ∈ {0;1} and *X* ∈ { +;−}. This order must be random because there is no way of gaining this information. Thus, maximum information to gain in this strategy is *I*_max_ = 2 instead of *I*_max_ = 3 when the order is known. Depending on the measurement outcomes, with probability $\tfrac{1}{2}$ the attacker can exclude some encodings and can guess the order of bases correctly only in half of the cases. Only if successful, half of 4 encodings can be eliminated. This makes ${I}_{\sec }=\tfrac{1}{4}{I}_{{\rm{\max }}}=\tfrac{1}{2}$.

## Conclusion and Discussion

We have successfully attacked a QM scheme based on QRG^8^. This scheme has been implemented in a form of quantum credit card containing quantum tokens. We retrieved the secret number (salt) used for preparing quantum tokens purely by means of imperfect quantum cloning and computational analysis of measured data (see Figs. 4 and 5). By learning the exact algorithm for encoding quantum tokens, the attacker is, in principle, able to produce perfect quantum money counterfeits. It is worth noting that the optimal strategy of our attack depends mainly on a particular implementation of bank’s security tolerances (e.g., losses) and chosen physical platform for implementing the attack. For instance, if the attacker uses deterministic optimal cloning even less qubit pairs is needed to perform the attack (see Fig. 2).

However, the attack was feasible because the bank encoded sufficiently high number of photon pairs using the same secret number (salt) and the same hash function. From the data summarised in Table 1 we can deduce that if the bank changes, e.g., the secret number after less then 1000 photon pairs, the attacker is not able to reveal the bank’s secret with sufficient certainty. This leads to further vital questions regarding tolerance of the bank to noise and threshold value losses.

We hope that our results will stimulate further research on security of QM schemes based on QRG bringing this concept closer to becoming a fully fledged quantum technology. Our results indicate that the correct secret number and hash function can be revealed assuming the hash function is a member of a finite set. The size of which is limited by the available time and computing power. However, this is not a fundamental limitation which might be lifted if more advanced cryptanalysis or more computing power is applied. Our results indicate that while the idea of using hash functions might be tempting, it would be ultimately more secure to store truly random sequences since only these are not vulnerable to the attack described in this paper. The recent progress in data storage technologies and quantum computing with its fast searching algorithms (e.g. Deutsch-Jozsa algorithm^39^) may in future enable this. With current technology, the most secure strategy would depend on particular implementation of the protocol by the bank.

## Supplementary information

Experimentally attacking quantum money schemes based on quantum retrieval games: Supplementary Material
