# Supplementary material for: Experimentally attacking quantum money schemes based on quantum retrieval games
Source: Sci Rep. 2019 Nov 8;9:16318. doi: 10.1038/s41598-019-51953-9 (PMC6841968; doi:10.1038/s41598-019-51953-9)
Supplement: Supplementary file 1 — Experimentally attacking quantum money schemes based on quantum retrieval games: Supplementary Material [file 41598_2019_51953_MOESM1_ESM.pdf]

Experimentally attacking quantum money schemes based on quantum retrieval games: Supplementary Material

Kateřina Jiráková,[1,*] Karol Bartkiewicz,[1,2,†] Antonín Černoch,[3,‡] and Karel Lemr[1,§]

[1]*RCPTM, Joint Laboratory of Optics of Palacký University and Institute of Physics of Czech Academy of Sciences, 17. listopadu 12, 771 46 Olomouc, Czech Republic*[¶]

[2]*Faculty of Physics, Adam Mickiewicz University, PL-61-614 Poznań, Poland*

[3]*Institute of Physics of the Czech Academy of Sciences, Joint Laboratory of Optics of PU and IP AS CR, 17. listopadu 50A, 772 07 Olomouc, Czech Republic*

In this supplement we give more technical details on deriving some expressions used in the main text and their explicit form.

STRATEGIES FOR ASSIGNING INFORMATION TO MEASUREMENTS PERFORMED ON CLONED PAIRS

During the transaction, a pair of states is taken from the card, which the hacker clones and from which we receive two pairs of copied qubits. To carefully consider the actual attack it is necessary to present all the situations in which the states are distorted. Using the knowledge of the protocol and assuming high fidelity of cloning, we can consider three strategies applied for analysing the results of measurements made on cloned pairs. For each measurement result, we assume the most likely situation.

Strategy a

We assume that both pairs of qubits are cloned perfectly. The measurements result in the same result for clones of the first qubit from a given pair and different results for clones of the second qubit from the pair. This is a strategy that allows to reject the largest number of possible options and gives the most information about the cloned state
Example:

- The hacker measures two pairs $(|VA\rangle, |VA\rangle)$ in the same basis, i.e., Q_{zz}.
- The result is: $(|VH\rangle, |VV\rangle)$ or $(|VV\rangle, |VH\rangle)$.
- The last bit of the hacker's information is assigned at random: $(01r)$.

Strategy b

We assume that both qubits are cloned ideally, but the measurement results on each pair of clones are the same. This is a strategy allows us to reject half of the possible options, from which we are still able to obtain some information about the cloned state.
Example:

- The hacker measures two pairs $(|VA\rangle, |VA\rangle)$ in the same basis, i.e., Q_{zz}.
- The result is: $(|VH\rangle, |VH\rangle)$ or $(|VV\rangle, |VV\rangle)$.
- The second or the third bit of the hacker's information is information is random: $(01r \,\text{or}\, 1r0) = (010, 011)$ or $(100, 110)$, or $(01r \,\text{or}\, 1r1) = (010, 011)$ or $(101, 111)$. This results in four random options.

Strategy c

We assume that one of the clones is orthogonal to the cloned state. This is a strategy that does not allow the elimination of any possibility. We do not get any information about the cloned state.
Example:

- The hacker measures two pairs $(|HA\rangle, |VA\rangle)$ in the same basis Q_{zz}.
- The result is: $(|HH\rangle, |VV\rangle)$ or $(|HV\rangle, |VH\rangle)$.
- All bits of the hacker's information are random, i.e., 8 options are equally probable: $(000, 001, 010, 100, 110, 101, 011, 111)$.

Additional variants

There is also an option that two or more clones end up in the orthogonal state resulting in errors in the hacker's information. The probability of such situations is, however, for optimal phase-covariant cloner no larger than $(F-1)^2 = 0.0213$. In Tab. I we give the probability of a successful attack on a single pair when both qubits from a pair are cloned (variant 1) for strategies a_1 and b_1. The probability of a successful attack (assigning the correct information to a pair of qubits) for a single pair for measurement in any basis, when both qubits in the pair are cloned for strategy c (i.e., strategy c_1) is constant and equals to $\frac{1}{8}(F-1)F$. In Tab. I we also present

the probabilities for the case where only one qubit from the pair has been cloned (variant 2, strategies a_2 and b_2). The probability of a successful attack on a single pair for measurement in any basis for strategy c when only one qubit from a pair is cloned (i.e., variant 2, strategy c_2) is constant and equals to $\frac{1}{16}(F - F^2 + \frac{1}{4})$. Note that very similar analysis is valid for measurements Q_{xx}.

The table has been created using the following procedure. We assume that if the bank sends a bit sequence, let it be 000 (in general it is X − 8 possible sequences). Next, the cloning is performed. With probability P^2 it succeeds twice, with probability $2(1 - P)P$ it succeeds once with only one of two qubits in the sequence, and with probability $(1 - P)^2$ it fails and the attacker learns nothing. At this point we have already 4 cases to consider for 8 inputs. To simplify our explanations, let us analyze a case where $X = 000 \longrightarrow |HD\rangle$ (the same procedure is applied for all 8 inputs).

- **Variant 0:** With probability $(1 - P)^2$ neither of the clones is created, thus, as in the case of erasure channel we get the following direct product of two probabilistic spaces: $\{[|HD\rangle, 1/4], [|HA\rangle, 1/4], [|VD\rangle, 1/4], [|VA\rangle, 1/4]\} \times \{[|HD\rangle, 1/4], [|HA\rangle, 1/4], [|VD\rangle, 1/4], [|VA\rangle, 1/4]\}$.
- **Variant 1:** For two clones the outcome of optimal cloning appearing with probability P^2 is a direct product of two probabilistic spaces, i.e., with probability P^2 both clones are created and F is fidelity of cloning: $\{[|HD\rangle, F^2], [|HA\rangle, F(1 - F)], [|VD\rangle, F(1 - F)], [|VA\rangle, (1 - F)^2]\} \times \{[|HD\rangle, F^2], [|HA\rangle, F(1 - F)], [|VD\rangle, F(1 - F)], [|VA\rangle, (1 - F)^2]\}$.
- **Variant 2:** With probability $(1 - P)P$ the first qubit is cloned resulting in the following direct product of two probabilistic spaces: $\{[|HD\rangle, \frac{F}{2}], [|HA\rangle, \frac{F}{2}], [|VD\rangle, \frac{1-F}{2}], [|VA\rangle, \frac{1-F}{2}]\} \times \{[|HD\rangle, \frac{F}{2}], [|HA\rangle, \frac{F}{2}], [|VD\rangle, \frac{1-F}{2}], [|VA\rangle, \frac{1-F}{2}]\}$
- **Variant 2:** With probability $(1 - P)P$ the second qubit is cloned resulting in the following direct product of two probabilistic spaces: $\{[|HD\rangle, \frac{F}{2}], [|HA\rangle, \frac{1-F}{2}], [|VD\rangle, \frac{F}{2}], [|VA\rangle, \frac{1-F}{2}]\} \times \{[|HD\rangle, \frac{F}{2}], [|HA\rangle, \frac{1-F}{2}], [|VD\rangle, \frac{F}{2}], [|VA\rangle, \frac{1-F}{2}]\}$.

To complete the stochastic trees we need to explain the decision process of the attacker as outlined above to guess three bits Y according to strategies a,b, and c. Let us choose query Q_{xx}. The attacker can measure (with some probability given by the above-listed probabilistic spaces):

- $DD, DD \longrightarrow$ set $Y = 000$ or $Y = 001$ or $Y = 101$ or $Y = 110$ with equal probability.
- $DD, DA \longrightarrow$ set $Y = 000$ or $Y = 001$ with equal probability.
- $DD, AD \longrightarrow$ set $Y = 100$ or $Y = 101$ with equal probability.
- $DD, AA \longrightarrow$ impossible, set any Y with equal probability OR do nothing.
- $DA, DD \longrightarrow$ set $Y = 000$ or $Y = 001$ with equal probability.
- $DA, DA \longrightarrow$ set $Y = 001$ or $Y = 011$ or $Y = 100$ or $Y = 101$ with equal probability
- $DA, AD \longrightarrow$ impossible, set any Y with equal probability OR do nothing.
- $DA, AA \longrightarrow$ set $Y = 001$ or $Y = 011$ with equal probability.
- $AD, DD \longrightarrow$ set $Y = 000$ or $Y = 010$ with equal probability.
- $AD, DA \longrightarrow$ impossible, set any Y with equal probability OR do nothing.
- $AD, AD \longrightarrow$ set $Y = 110$ or $Y = 111$ or $Y = 000$ or $Y = 010$ with equal probability.
- $AD, AA \longrightarrow$ set $Y = 110$ or $Y = 111$ with equal probability.
- $AA, DD \longrightarrow$ impossible, set any Y with equal probability OR do nothing.
- $AA, DA \longrightarrow$ set $Y = 001$ or $Y = 011$ with equal probability.
- $AA, AD \longrightarrow$ set $Y = 110$ or $Y = 111$ with equal probability.
- $AA, AA \longrightarrow$ set $Y = 001$ or $Y = 011$ or $Y = 110$ or $Y = 111$ with equal probability.

If the query is Q_{zz}, the logic is the same. Finally, by tracking the relevant branches of stochastic tree, we create probability tables. Having explained all the steps, a complete stochastic tree for a given query would have the following structure: 8 input states $X \longrightarrow 4$ cloning failure/success events $\longrightarrow 16$ qubit pairs $\longrightarrow 16$ measurement outcomes $\longrightarrow 8$ states Y. The complete analysis of the tree would correspond to tracking 8 nodes $\longrightarrow$ 32 nodes $\longrightarrow$ 512 nodes $\longrightarrow$ 8192 nodes $\longrightarrow$ 65536 nodes. Note that the attacker knows when the cloning succeeds/fails and what is measured. Thus, also for each cloning failure/success event and each query a separate probability table is created. Finally, three different probability tables are created depending on the decision strategy (the attacker differentiates between the strategies).

The hacker, collecting the results of measurements, is able to learn the algorithm of encoding pairs of qubits. However, in order to make it possible the cloning fidelity must be optimized. Cloning operation inevitably involves

TABLE I. Joint probability distribution describing encoded bits and hacker's knowledge Y gained from an attack on a single pair of qubits encoding 3-bit sequence X for query Q_{zz} or after swapping two last bits of X and Y for query Q_{xx}. When cloning both qubits in the pair and deal with strategy a (i.e., strategy a_1), we have $p_1 = \frac{1}{4}F^2$, $p_2 = \frac{1}{4}(1-F)^2$, and $p_3 = \frac{1}{4}F(1-F)$. In the same regime, for strategy b (i.e., strategy b_1), we have $p_1 = \frac{1}{8}F^2$, $p_2 = \frac{1}{8}(1-F)^2$, and $p_3 = \frac{1}{8}F^2 - \frac{1}{8}F + \frac{1}{16}$. If successful cloning was achieved only with one qubit from a pair, we assume that the second qubit of the pair is associated with two completely mixed clones of the fidelity of 1/2. In this second regime we have for strategy a (i.e., strategy a_2) $p_1 = \frac{1}{8}F^2 + \frac{1}{32}$, $p_2 = \frac{1}{8}F^2 - \frac{1}{4}F + \frac{5}{32}$, and $p_3 = -\frac{1}{8}F^2 + \frac{1}{8}F + \frac{1}{32}$. Under the same assumption on cloning in case of strategy b we have $p_1 = \frac{1}{16}F^2 + \frac{1}{64}$, $p_2 = \frac{1}{16}F^2 - \frac{1}{16}F + \frac{5}{64}$, and $p_3 = \frac{1}{16}F^2 - \frac{1}{16}F + \frac{3}{64}$.

	000	001	010	011	100	101	110	111
000	p_1	p_1	p_2	p_2	p_3	p_3	p_3	p_3
001	p_1	p_1	p_2	p_2	p_3	p_3	p_3	p_3
010	p_2	p_2	p_1	p_1	p_3	p_3	p_3	p_3
011	p_2	p_2	p_1	p_1	p_3	p_3	p_3	p_3
100	p_3	p_3	p_3	p_3	p_1	p_1	p_2	p_2
101	p_3	p_3	p_3	p_3	p_1	p_1	p_2	p_2
110	p_3	p_3	p_3	p_3	p_2	p_2	p_1	p_1
111	p_3	p_3	p_3	p_3	p_2	p_2	p_1	p_1

causing errors in the measurement results used for verification. If the level of incorrect results exceeds the specified limit the transaction will be rejected. In order to minimize the error rate it is, therefore, necessary to implement an attack strategy that takes into account all measurement circumstances.

ATTACK-VERIFICATION SCENARIOS

There are 3 attack–verification scenarios that we consider in our work:

- **Scenario (i):** Providing the bank with results each time cloning takes place. If cloning fails, sending random values.
- **Scenario (ii):** Providing the bank with results only when the measurement is recorded by the terminal. In case of unsuccessful cloning, the loss of the qubit is reported.
- **Scenario (iii):** Measurement of qubits in the specified database after the card is removed from terminal, without cloning operation. Random results are sent to the bank.

Note that in the main text these cases are referred to as strategies. However, here it is more suitable to call them scenarios.

From direct calculations based on the probabilities leading to verification error, we can derive an expression concerning the frequency of errors in the verification of a pair of qubits ϵ. This is the probability of reporting an error to the bank. Note that it depends only on what happens to a qubit measured in a compatible basis. For each strategy, the error rate is described by the respective equation [see Eq. (2) and (3) in the main text], i.e.:

$$\epsilon_{\text{(i)}} = P(1-F) + (1-P)/2,$$
$$\epsilon_{\text{(ii)}} = (1-F),$$
$$\epsilon_{\text{(iii)}} = \frac{1}{2}.$$

The parameter $\epsilon_{(i)}$ takes into account two situations. In the first case, one or both cubits are lost during cloning and therefore random results are reported to the bank (50% chance of getting an error). In the second case, even if the cloning is successful, imperfect fidelity may cause the measurement to give an incorrect result. The error rate in scenario (ii) depends only on the imperfect fidelity of the cloning.

MUTUAL INFORMATION

In order to quantify the correlation between the attacker and the information encoded as a pair of qubits, we enter the value of mutual information I. This value determines how many bits of information an attacker can get after cloning one pair of qubits and depends on the strategy used, cloning the probability of success P and fidelity F. Mutual information is calculated as

$$I = I_{X,Y} = I_{Y,X} = \sum_{X,Y=000}^{111} p_{X,Y} \log_2 \frac{p_{X,Y}}{p_X p_Y},$$

where $p_X = \sum_{Y=000}^{111} p_{X,Y}$, $p_Y = \sum_{X=000}^{111} p_{X,Y}$, and $X, Y = 000, 001, 010, 100, 110, 101, 011, 111$. When considering scenario (i) to calculate mutual information we need to utilise probabilities from Tabs. 1–4 and possibility uniform probability distribution (the cloned pair is lost) referred to as strategy 0. The mutual information for security analysis of scenarios (i) and (ii), respectively, reads

$$I_{\text{sec(i)}} = P^2(I_{a_1} + I_{b_1} + I_{c_1}) + 2P(1-P)(I_{a_2} + I_{b_2} + I_{c_2}) + (1-P)^2 I_0$$

and

$$I_{\text{sec(ii)}} = I_{a_1} + I_{b_1} + I_{c_1},$$

where $I_0 = 0$ and the subscripts denote the strategy. These values are query independent. For scenario (iii) the information learned by the hacker is $I_{\text{sec(iii)}} = \frac{1}{2}$.

TABLE II. Joint probability distribution describing encoded bits and hacker's knowledge Y gained from an attack on a single pair of qubits encoding 3-bit sequence X for query Q_{xx} (i.e., XX–basis measurement) for scenario (iii), where the attacker assumes at random encoding XZ or ZX.

	000	001	010	011	100	101	110	111
000	$\frac{1}{32}$	0	$\frac{1}{32}$	0	$\frac{1}{64}$	$\frac{1}{64}$	$\frac{1}{64}$	$\frac{1}{64}$
001	0	$\frac{1}{32}$	0	$\frac{1}{32}$	$\frac{1}{64}$	$\frac{1}{64}$	$\frac{1}{64}$	$\frac{1}{64}$
010	$\frac{1}{32}$	0	$\frac{1}{32}$	0	$\frac{1}{64}$	$\frac{1}{64}$	$\frac{1}{64}$	$\frac{1}{64}$
011	0	$\frac{1}{32}$	0	$\frac{1}{32}$	$\frac{1}{64}$	$\frac{1}{64}$	$\frac{1}{64}$	$\frac{1}{64}$
100	$\frac{1}{64}$	$\frac{1}{64}$	$\frac{1}{64}$	$\frac{1}{64}$	$\frac{1}{32}$	0	$\frac{1}{32}$	0
101	$\frac{1}{64}$	$\frac{1}{64}$	$\frac{1}{64}$	$\frac{1}{64}$	0	$\frac{1}{32}$	0	$\frac{1}{32}$
110	$\frac{1}{64}$	$\frac{1}{64}$	$\frac{1}{64}$	$\frac{1}{64}$	$\frac{1}{32}$	0	$\frac{1}{32}$	0
111	$\frac{1}{64}$	$\frac{1}{64}$	$\frac{1}{64}$	$\frac{1}{64}$	0	$\frac{1}{32}$	0	$\frac{1}{32}$

TABLE III. Joint probability distribution describing encoded bits and hacker's knowledge Y gained from an attack on a single pair of qubits encoding 3-bit sequence Z for query Q_{zz} (i.e., ZZ–basis measurement) for scenario (iii), where the attacker assumes at random encoding XZ or ZX.

	000	001	010	011	100	101	110	111
000	$\frac{1}{32}$	$\frac{1}{32}$	0	0	$\frac{1}{64}$	$\frac{1}{64}$	$\frac{1}{64}$	$\frac{1}{64}$
001	$\frac{1}{32}$	$\frac{1}{32}$	0	0	$\frac{1}{64}$	$\frac{1}{64}$	$\frac{1}{64}$	$\frac{1}{64}$
010	0	0	$\frac{1}{32}$	$\frac{1}{32}$	$\frac{1}{64}$	$\frac{1}{64}$	$\frac{1}{64}$	$\frac{1}{64}$
011	0	0	$\frac{1}{32}$	$\frac{1}{32}$	$\frac{1}{64}$	$\frac{1}{64}$	$\frac{1}{64}$	$\frac{1}{64}$
100	$\frac{1}{64}$	$\frac{1}{64}$	$\frac{1}{64}$	$\frac{1}{64}$	$\frac{1}{32}$	$\frac{1}{32}$	0	0
101	$\frac{1}{64}$	$\frac{1}{64}$	$\frac{1}{64}$	$\frac{1}{64}$	$\frac{1}{32}$	$\frac{1}{32}$	0	0
110	$\frac{1}{64}$	$\frac{1}{64}$	$\frac{1}{64}$	$\frac{1}{64}$	0	0	$\frac{1}{32}$	$\frac{1}{32}$
111	$\frac{1}{64}$	$\frac{1}{64}$	$\frac{1}{64}$	$\frac{1}{64}$	0	0	$\frac{1}{32}$	$\frac{1}{32}$

Note that for this strategy while the attacker can eliminate some of 8 encodings (values of Y), these eliminated encodings depend on the order of basis. The attacker can assume/guess that the order of encoding bases for the received pair of qubits is XZ or ZX. The order must be random because there is no way of gaining this information (thus maximum information to gain is here $I_{\max} = 2$ instead of $I_{\max} = 3$ when the order is known). Then, under this assumption, with probability 1/2 the attacker, depending on the measurement outcomes (query Q_{xx} or Q_{zz} - honest but curious attacker), can exclude some encodings. The attacker can guess the order of bases correctly only in half of the cases. Only if successful, half of 4 encodings can be eliminated. This makes $I_{\rm sec(iii)} = \frac{1}{4} I_{max} = \frac{1}{2}$. This is confirmed by direct calculations based on Tab. II or Tab. III.

Note that Figs. 2 and 3 presented in the main text are depict functions $I_{sec(n)}[\epsilon(n)]$ for $n = \mathrm{i}, \mathrm{ii}, \mathrm{iii}$. In case of Fig. 3 depicting conditional mutual information, $I_{sec(i)}$ is calculated assuming $P = 1$, because in this case the hacker infers the information only if both qubits are cloned.

* katerina.jirakova@upol.cz

† bark@amu.edu.pl

‡ acernoch@fzu.cz

§ k.lemr@upol.cz

¶ Presently on leave at Faculty of Physics, Adam Mickiewicz University, PL-61-614 Poznań, Poland
